# Understanding the heterogeneity in liver hepatocellular carcinoma with a special focus on malignant cell through single-cell analysis

**DOI:** 10.1007/s12672-024-01115-9

**Published:** 2024-06-24

**Authors:** Mengmeng Bao, Anshi Wu

**Affiliations:** Department of Anesthesiology, Beijing Chaoyang Hospital, Capital Medical University, Beijing, 100020 China

**Keywords:** Hepatocellular carcinoma, Prognosis, Intratumor heterogeneity, Immune landscape

## Abstract

**Introduction:**

Hepatocellular carcinoma (HCC) is the most common form of liver cancer globally and remains a major cause of cancer-related deaths. HCC exhibits significant intra-tumoral and interpatient heterogeneity, impacting treatment efficacy and patient prognosis.

**Methods:**

We acquired transcriptome data from the TCGA and ICGC databases, as well as liver cancer chip data from the GEO database, and processed the data for subsequent analysis. We also obtained single cell data from the GEO database and performed data analysis using the Seurat package. To further investigate epithelial cell subgroups and their copy number variations, we used the Seurat workflow for subgroup classification and the InferCNV software for CNV analysis, utilizing endothelial cells as a reference. Pseudo-time analysis and transcription factor analysis of epithelial cells were performed using the monocle2 and SCENIC software, respectively. To assess intercellular communication, we employed the CellChat package to identify potential ligand-receptor interactions. We also analyzed gene expression differences and conducted enrichment analysis using the limma and clusterProfiler packages. Additionally, we established tumor-related risk characteristics using Cox analysis and Lasso regression, and predicted immunotherapy response using various datasets.

**Results:**

The samples were classified into 23 clusters, with malignant epithelial cells being the majority. Trajectory analysis revealed the differentiation states of the malignant epithelial cells, with cluster 1 being in the terminal state. Functional analysis revealed higher aggressiveness and epithelial-mesenchymal transition (EMT) scores in cluster 1, indicating a higher propensity for metastasis. RBP4+ tumor cells were highly enriched with hypoxia process and intensive cell-to-cell communication. A prognostic model was established, and immune infiltration analysis showed increased infiltration in the high-risk group. TP53 demonstrated significant differences in mutation rate between the two risk groups. Validation analysis confirmed the up-regulation of model genes, including AKR1B10, ARL6IP4, ATP6V0B, and BSG in tumor tissues.

**Conclusion:**

A prognostic model was established based on HCC malignant cell associated gene signature, displaying decent prognosis guiding effectiveness in the multiple cohorts. The study provided comprehensive insights into the heterogeneity and potential therapeutic targets of LIHC.

## Introduction

Liver hepatocellular carcinoma (LIHC), known as hepatocellular carcinoma (HCC), is the most prevalent form of primary liver cancer worldwide, constituting over 80% of cases. Despite advancements in diagnosis and treatment, liver cancer remains the sixth most common cancer globally and the fourth leading cause of cancer-related deaths [1]. Although the age-standardized incidence rate of liver cancer is declining, the overall burden of the disease continues to rise, with a low 5-year survival rate of only 12% [2]. Researchers have made significant strides in understanding the development mechanisms of HCC and characterizing its heterogeneity [3]. Muti-Omics analysis was widely implemented to study innovative therapeutic strategies to address this pressing clinical issue across various cancers [4, 5]. However, there is still much to be learned about the immune dysfunction underlying HCC and its potential as a therapeutic target.

HCC exhibits significant heterogeneity—both within tumors and across patients [6]. While the latter is crucial for personalized therapy, the former, known as intra-tumoral heterogeneity (ITH), can significantly impact treatment efficacy on an individual level [7]. ITH is a key contributor to tumor growth, metastasis, recurrence, and drug resistance, and ultimately hampers patient prognosis [8]. Therefore, there is a pressing need to comprehensively understand the underlying causes, characteristics, and implications of tumor heterogeneity in HCC, which can guide clinical practice and ultimately improve patient survival.

In this study, by analyzing transcriptome data from various databases and performing comprehensive analyses, we sought to shed light on the immune landscape of HCC and identify potential targets for immunotherapy. The samples were classified into 23 clusters, with the majority being malignant epithelial cells. Trajectory analysis revealed the differentiation states of these cells, with cluster 1 being in the terminal state. Functional analysis showed that cluster 1 had higher aggressiveness and EMT scores. RBP4+ tumor cells were enriched in hypoxia processes and intensive cell-to-cell communication. A prognostic model was established, and immune infiltration analysis revealed increased infiltration in the high-risk group. TP53 had significant differences in mutation rate between the two risk groups. Validation analysis confirmed the up-regulation of model genes in tumor tissues. Overall, this study provided comprehensive insights into the heterogeneity and potential therapeutic targets of LIHC, and established a prognostic model with decent prognosis guiding effectiveness.

## Methods

### Acquisition and processing of transcriptome data

The transcriptome data for LIHC was obtained from the TCGA database, consisting of clinical data of 368 patients. This dataset was used as the training group to construct our model. All transcriptome data was transformed into Transcripts Per Million (TPM) format and then log_2_ transformed for subsequent analyses. Additionally, the LIRI-JP dataset from the ICGC database, consisting of 232 samples, was used as the verification group. The GSE14520 (T = 225, N = 220) and GSE45267 (T = 48, N = 39) LIHC chip data from the GEO database were included in the set of differential gene analysis. The normalizeBetweenArrays function in the limma package was employed to correct for any variations in the chip data. Furthermore, the Combat algorithm from the sva package was utilized for the correction of different batches of data.

### Acquisition and processing of single cell data

The single-cell dataset used in this study was obtained from GSE149614 in the GEO database, which comprised 10 primary tumor samples. Data analysis was conducted using R software (version 4.1.3), with the Seurat package used for analysis. Only cells with a mitochondrial content of less than 20% under the condition of cytoplasmic control were included in the analysis. The UMI count and gene count were limited to a range of 200–50,000 and 200–8000, respectively. Data normalization, selection of highly variable genes (2000), and data transformation [to eliminate the effect of the cell cycle, utilizing the parameter vars.to.regression = c(‘S.Score’, ‘G2M.Score’)] was performed using the NormalizeData, FindVariableFeatures, and ScaleData functions from the Seurat package. To account for batch effects, the harmony algorithm was applied. Dimensionality reduction techniques, such as UMAP and tSNE, and the Louvain clustering algorithm, were employed using the corresponding functions provided by the Seurat package. Differential gene expression analysis was conducted using the FindAllMarkers function, with parameters set to p-value < 0.05, log_2_ fold change > 0.25, and an expression ratio greater than 0.1.

### Cell annotation analysis

To characterize the cell types present in our dataset, we employed specific marker genes for epithelial cells (‘EPCAM’, ‘KRT18’, ‘KRT19’, ‘CDH1’), fibroblasts (‘DCN’, ‘THY1’, ‘COL1A1’, ‘COL1A2’), endothelial cells (‘PECAM1’, ‘CLDN5’, ‘FLT1’, ‘RAMP2’), T cells (‘CD3D’, ‘CD3E’, ‘CD3G’, ‘TRAC’), NK cells (‘NKG7’, ‘GNLY’, ‘NCAM1’, ‘KLRD1’), B cells (‘CD79A’, ‘IGHM’, ‘IGHG3’, ‘IGHA2’), and mast cells (‘KIT’, ‘MS4A2’, ‘GATA2’). Based on these markers, we performed separate clustering analyses for epithelial cells to explore tumor heterogeneity. Various charts, including UMAP, tSNE, histograms, and heat maps, were generated to visualize the results.

### Subgroup and CNV analysis of epithelial cells

To further dissect the subgroups within the epithelial cell population, we conducted a separate analysis using the standard Seurat workflow. Subgroup classification was based on the copy number variation (CNV) score of each subgroup, with TSNE plots used for visualization. Additionally, to investigate CNVs within the tumor and epithelial cell subsets, we employed the InferCNV software. This analysis, using endothelial cells as a reference, aimed to identify malignant cells and evaluate the CNV scores of the different epithelial cell subgroups.

### Pseudo-time analysis of epithelial cells

We employed the monocle2 software to perform pseudo-time analysis of the epithelial cell subsets. The dimension reduction algorithm used was DDRTree, and default parameters were applied for the remaining steps. This analysis aimed to uncover the differentiation processes of these cells.

### Transcription factor analysis of epithelial cells

To investigate the involvement of transcription factors in the epithelial cell subsets, we utilized the SCENIC software. This analysis utilized the RcisTarget and GRNBoost motif databases with default parameters. The RcisTarget package was employed to identify overexpressed transcription factor binding motifs within the gene list. The AUCell software package was used to score the activity of each regulator group for each cell type.

### Cell-to-cell communication analysis

To assess potential intercellular communication, we employed the CellChat package. The CellChat function was used to import the normalized gene expression matrix and create the CellChat object. Subsequently, the identifyOverExpressedGenes, identifyOverExpressedInteraction, and ProjectData functions were utilized with default parameters for data preprocessing. The computeCommunProb, filterCommunication, and computeCommunProbPathway functions were employed to identify potential ligand-receptor interactions. Finally, the aggregateNet function was utilized to generate the cell communication network.

### Analysis of gene expression differences and enrichment analysis

The limma software was utilized to calculate the differential expression of genes between tumor and adjacent samples in the GEO and TCGA datasets. A significance threshold of p < 0.05 was applied to identify DEGs. The clusterProfiler package was then used to perform enrichment analysis on the up-regulated and down-regulated genes. The algorithm used was GSEA, and the functional databases utilized were Gene Ontology Biological Process (GOBP) and KEGG. The gene sets used to define functional signatures were sourced from the msigdb database. The results of the enrichment analysis were visualized using the enrichplot package.

### Establishment of tumor-related risk characteristics

First, the intersection of the differentially expressed genes in the epithelial cells, GEO dataset, and TCGA dataset was obtained to identify a set of genes relevant to tumor biology. Single-factor Cox analysis was then utilized to extract genes from this set that had prognostic value. Further screening of these prognostic genes was performed using Lasso regression to establish a prognostic model. This model assigns a risk score to each patient, which is used to stratify them into high-risk and low-risk groups based on the median score in the TCGA cohort. We used the “timeROC” package to draw Receiver Operating Characteristic Curve(ROC) to verify the testing efficiency of the model. The area under the curve (AUC) greater than 0.6 was considered to have good testing efficiency.

### Prediction of immunotherapy response

To predict the response to immunotherapy, datasets GSE35640 (melanoma), GSE91061 (melanoma), IMvigor210 (urothelial carcinoma, UC), and GSE126044 (non-small cell lung cancer, NSCLC) were collected. The risk model scores were calculated for each dataset, and these scores were used to predict the response to immunotherapy.

### Tumor immune infiltration analysis and tumor immunity profiling (TIP) analysis

We utilized the IOBR package to assess the extent of immune cell infiltration in LIHC patients from the TCGA database. We employed six evaluation methods, namely CIBERSORT, quanTIseq, MCPcounter, xCell, EPIC, and Estimate, to determine the immune cell composition within the TME. These results were utilized to generate a heat map, quantifying the relative abundance of immune cell infiltration in the TME. Additionally, the Estimate algorithm provided insights into the relative proportions of stromal cells, immune cells, and tumor cells, enabling comparisons across different risk categories. TIP analysis was conducted using the online URL (http://biocc.hrbmu.edu.cn/TIP/).

### Drug sensitivity and mutation analysis

We employed the ‘oncoPredict’ R package to calculate the IC50 of commonly used chemotherapeutic drugs. This analysis allowed us to evaluate the relationship between risk scores and drug sensitivity. The Wilcoxon rank sum test was employed to compare IC50 values between the two risk groups. Mutation data for LIHC were obtained from the TCGA GDC database, and analyzed using the maftools package. Visualization of the mutation data was performed using the oncoPrint function from the ComplexHeatmap package.

### Enrichment analysis of single-sample gene sets

To assess the enrichment score of gene sets in individual samples, we employed single-sample Gene Set Enrichment Analysis (ssGSEA). In this study, ssGSEA analysis was utilized to determine the risk score for each patient.

### Statistical analysis

All data processing, statistical analysis, and visualization were conducted using R 4.1.3 software. The correlation between continuous variables was assessed using the Pearson correlation coefficient. Categorical variables were compared using the chi-square test, while continuous variables were compared using the Wilcoxon rank sum test or t-test. Cox regression and Kaplan–Meier analysis were performed using the survival package. In this study, the standard of statistical significance p value was: p < 0.05 was considered to be statistically significant.

## Results

### Single-cell expression profile of LIHC samples

**Fig. 1:**
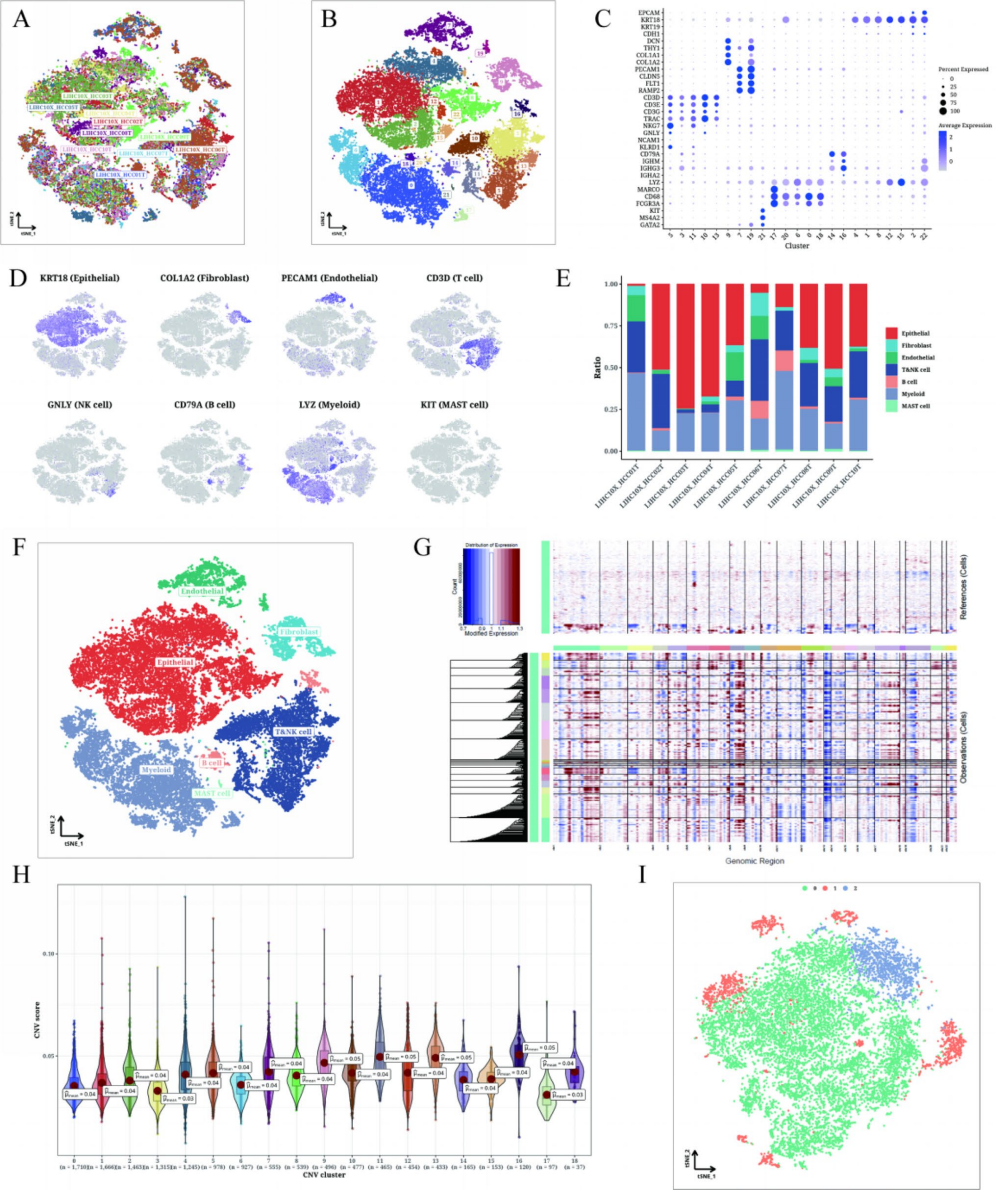
Single cell classification results of LIHC. **A**, **B** t-SNE plots colored according to sample and cluster classification. Clusters were determined based on a correlation map of marker gene expression (**C**). t-SNE plot (**D**) showing the expression of each marker gene. Histogram (**E**) indicating the cell types in each sample. t-SNE plot (**F**) displaying the cell annotation results. CNV heat map of epithelial cells (**G**) using endothelial cells as a reference. Violin plot (**H**) showing the CNV scores of epithelial cell clusters. t-SNE plot (**I**) classifying epithelial cells based on CNV scores

The study comprised 10 samples, each demonstrating a relatively stable cell distribution, suggesting minimal sensitivity to batch effects (Fig. 1A). By implementing the TSNE algorithm, all identified cells were classified into 23 clusters, allowing for detailed categorization (Fig. 1B). Figure 1C, D illustrated the expression pattern of each marker gene associated with the 23 cell clusters. To assess the distribution of these seven cell types across the 23 samples, malignant epithelial cells accounted for the majority of annotated cell population, followed by T cells and myeloid cells (Fig. 1E). Notably, the presence and distribution of all identified cell types, including epithelial cells, T cells, and fibroblasts (Fig. 1F). Furthermore, Fig. 1G portrayed the CNV of each chromosome. Notably, significant copy number loss was observed on chromosome 6 in nearly all tumor cells. To explore the distribution differences in CNV scores among the 19 clusters, Fig. 1H highlights clusters 9, 11, 13, and 16, characterized by increased CNV, and clusters 3 and 17, characterized by decreased copy number. Additionally, epithelial cells within these clusters underwent dimensionality reduction via TSNE, enabling their subdivision into three distinct subclusters: cluster 0, cluster 1, and cluster 2 (Fig. 1I).

### Trajectory analysis and cell communication analysis of epithelial cells

**Fig. 2:**
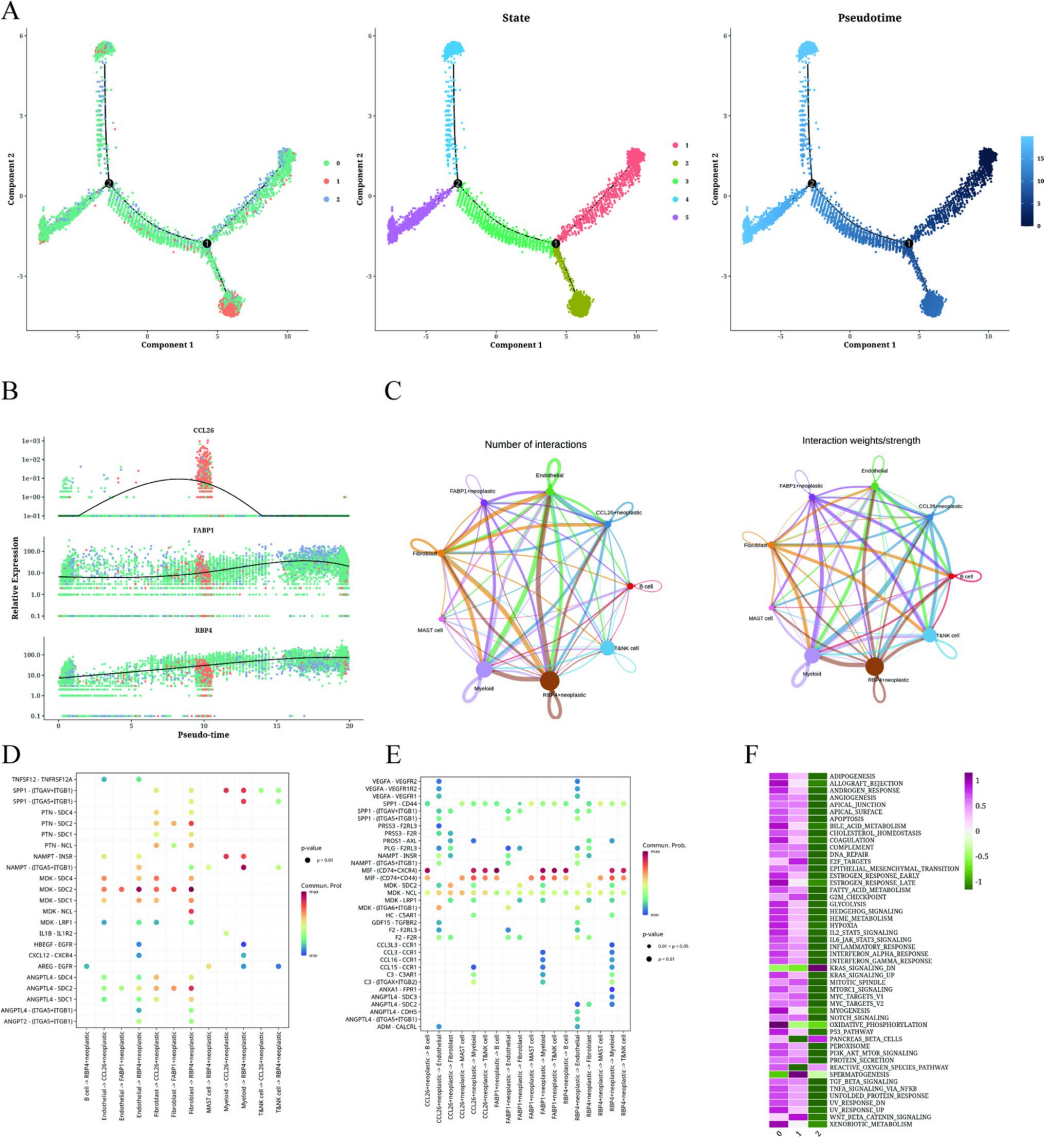
Trajectory analysis and cell communication analysis of epithelial cells. **A** Differentiation trajectory, quasi-time distribution, quasi-time cell clusters, and proportions of each cluster in all epithelial cells. **B** Pseudo-time expression of RBP4, CCL26, and FABP1. **C** Number and intensity of cell communication between RBP4+ tumor, CCL26+ tumor, FABP1+ tumor, and other cell types. Bubble plots (**D**) showing the interaction of RBP4+ tumor, CCL26+ tumor, and FABP1+ tumor with different cell ligand-receptor pairs. Ligand-receptor bubble diagrams (**E**) illustrating the interaction of different cell types with RBP4+ tumors, CCL26+ tumors, and FABP1+ tumors. **F** Enrichment analysis of three clusters

Using the ‘monocle2’ R software package, trajectory analysis was employed to assess the transcriptional heterogeneity of malignant epithelial cells (Fig. 2A). The pseudo-time progression revealed that the cluster 2 subtype was spread throughout the entire differentiation, while the cluster 0 subtype was in the intermediate state, and the cluster 1 subtype was at the terminal state. Figure 2B displayed the relative expression of the three most significantly altered genes, RBP4, CCL26, and FABP1, providing insights into the temporal dynamics of gene expression changes. CCL26 was highly expressed in the intermediated state, while FABP1 and RBP4 were increasingly expressed along the pseudo-time. Figure 2C illustrates the number and intensity of cell communication between RBP4+ tumor cells, CCL26+ tumor cells, FABP1+ tumor cells, and other cell types in the tissue. RBP4+ tumor cells displayed the highest communication pattern along different types of LIHC malignant cells, showing intensive interactions with the endothelial cells, myeloid cells and CAFs. Notably, all three tumor cell types were found to bind to other cell types via MDK-NCL receptor-ligand pairs (Figs. 2D and E). Furthermore, Fig. 2F evaluated the enrichment of the three identified cell clusters. RBP4+ and CCL26+ tumor cells demonstrated enrichment in nearly all pathways, indicating their prominence in multiple biological processes. Intriguingly, RBP4+ tumor cells were highly enriched with hypoxia process. Conversely, FABP1+ tumor cells primarily exhibited enrichment in KRAS_SIGNALING_DN.

### Transcription factor analysis of epithelial cells

**Fig. 3:**
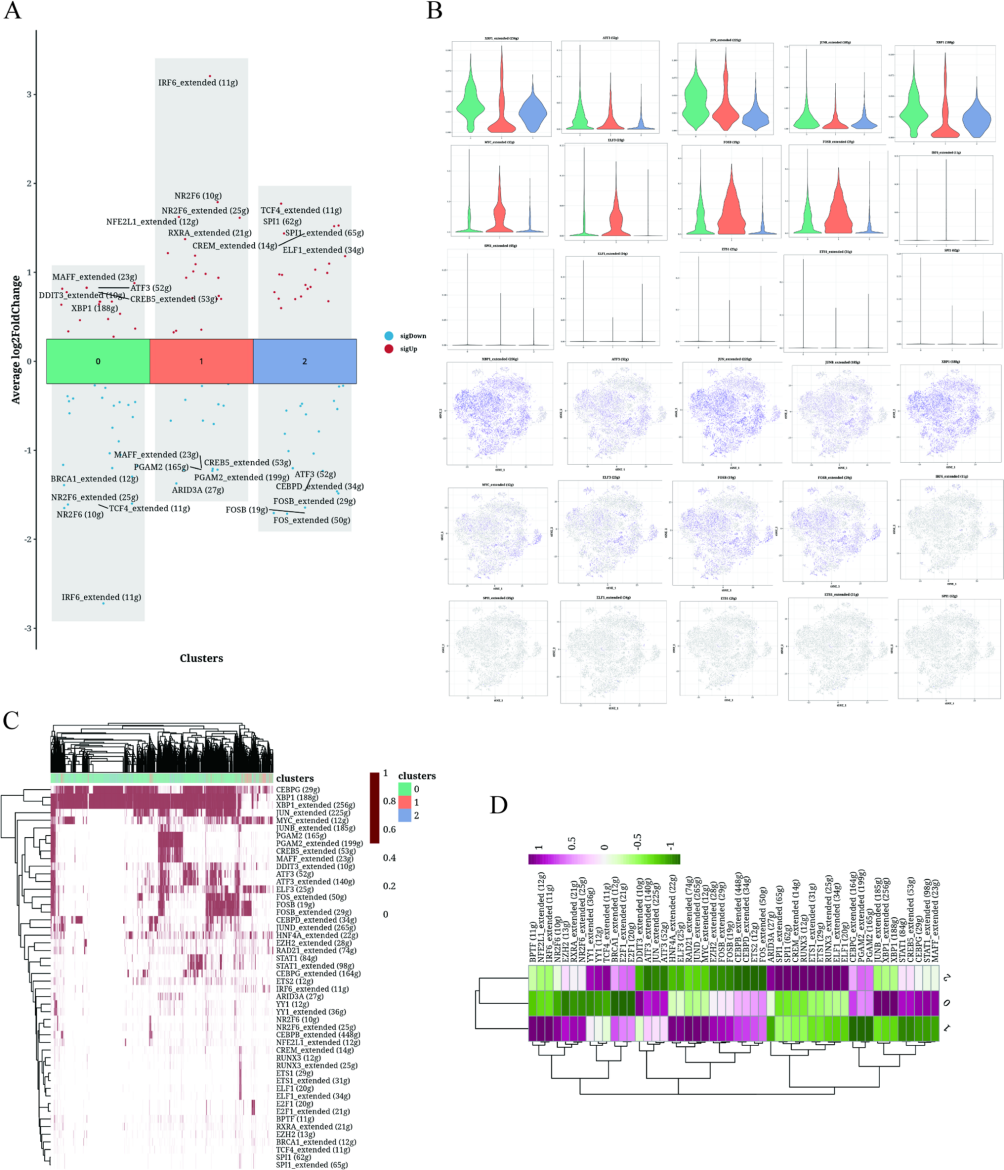
Transcription factor analysis results of epithelial cells. **A** Volcano plots displaying the highly expressed and lowly expressed genes in each cluster. **B** Violin plot and UMAP plot showing the expression of five genes in each cluster. **C**, **D** Heatmap depicting the distribution of gene regulatory elements in different clusters

Figure 3A displayed the top five highly expressed gene transcription factors and the top five lowly expressed gene transcription factors in each cell cluster. NR2F6 was highly enriched in the cluster1, while ranked at the lowest expression in the cluster 0. ELF3 and FOSB were shown to be exclusively expressed in the cluster 1 (Fig. 3B), while XBP1 showed an opposed pattern (Fig. 3C and D).

### Functional analysis of aggressive and EMT

**Fig. 4:**
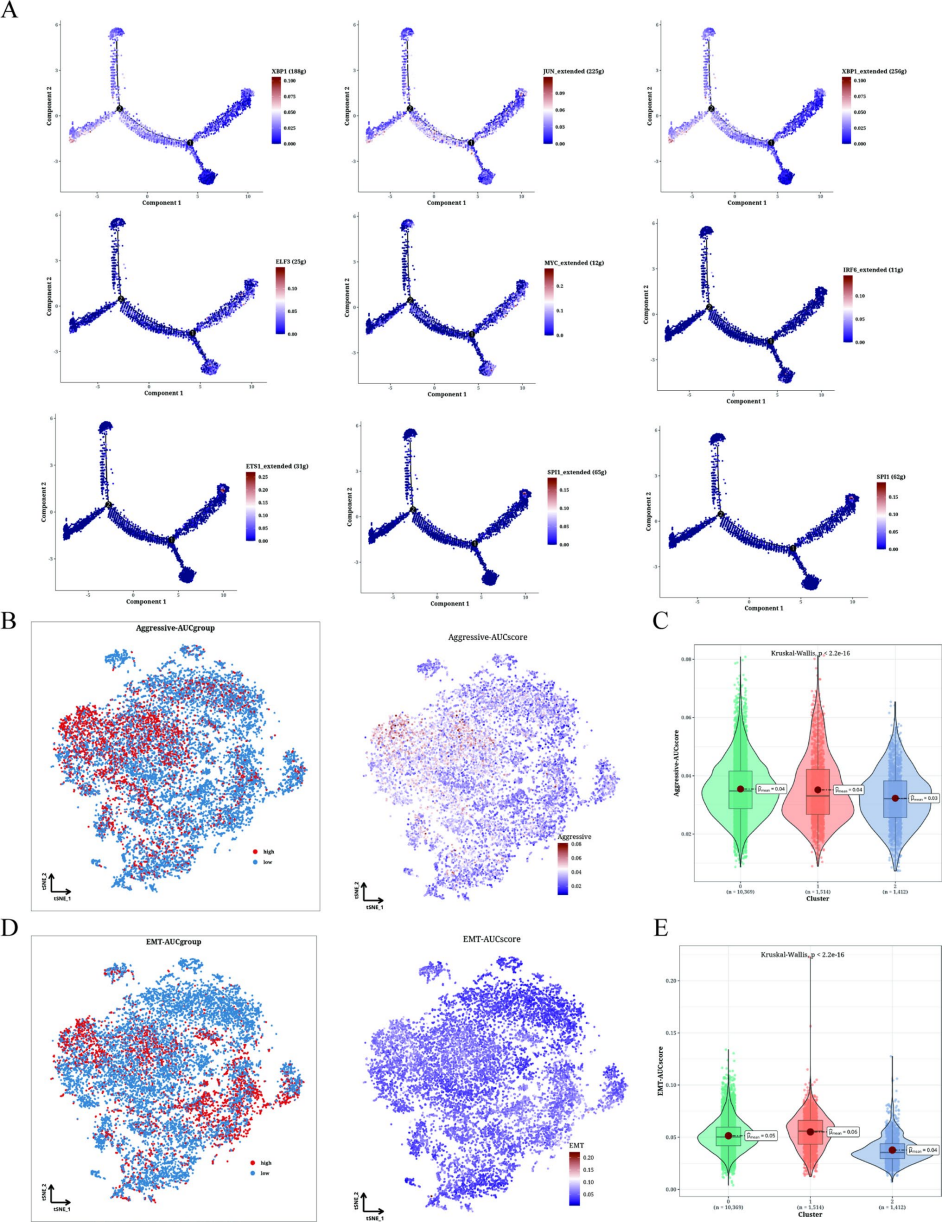
Functional analysis results of aggressiveness and EMT. **A** Cell trajectory analysis of different regulators. **B**, **C** Invasion levels of three clusters in t-SNE map and violin plot. **D**, **E** EMT levels of the three clusters in the t-SNE diagram and violin diagram

In Fig. 4A, we integrated the transcription factors with the highest specificity for the Cluster 0–2 epithelial cell subgroup into the pseudo-time reasoning analysis. Notably, JUN and XBP1 are up-regulated in cluster 0, while ELF3, IRF6, and MYC are up-regulated in cluster 1, and ETS1 and SPI1 are up-regulated in cluster 2. Figure 4B and C demonstrate that the Cluster 1 subgroup exhibits significantly higher Aggressive scores compared to the other cell subsets. This observation suggests an enhanced invasive ability of tumor cells within the Cluster 1 subgroup. Additionally, as depicted in Fig. 4D and E, there are significant differences in EMT scores between Cluster 0 and Cluster 1, 2. Specifically, Cluster 1 displays markedly higher EMT scores than Cluster 0 and Cluster 2, indicating a more pronounced migratory ability of LIHC epithelial cells within the Cluster 1 subgroup, which may be related to an increased propensity for metastasis.

### Prognostic model establishment and evaluation

**Fig. 5:**
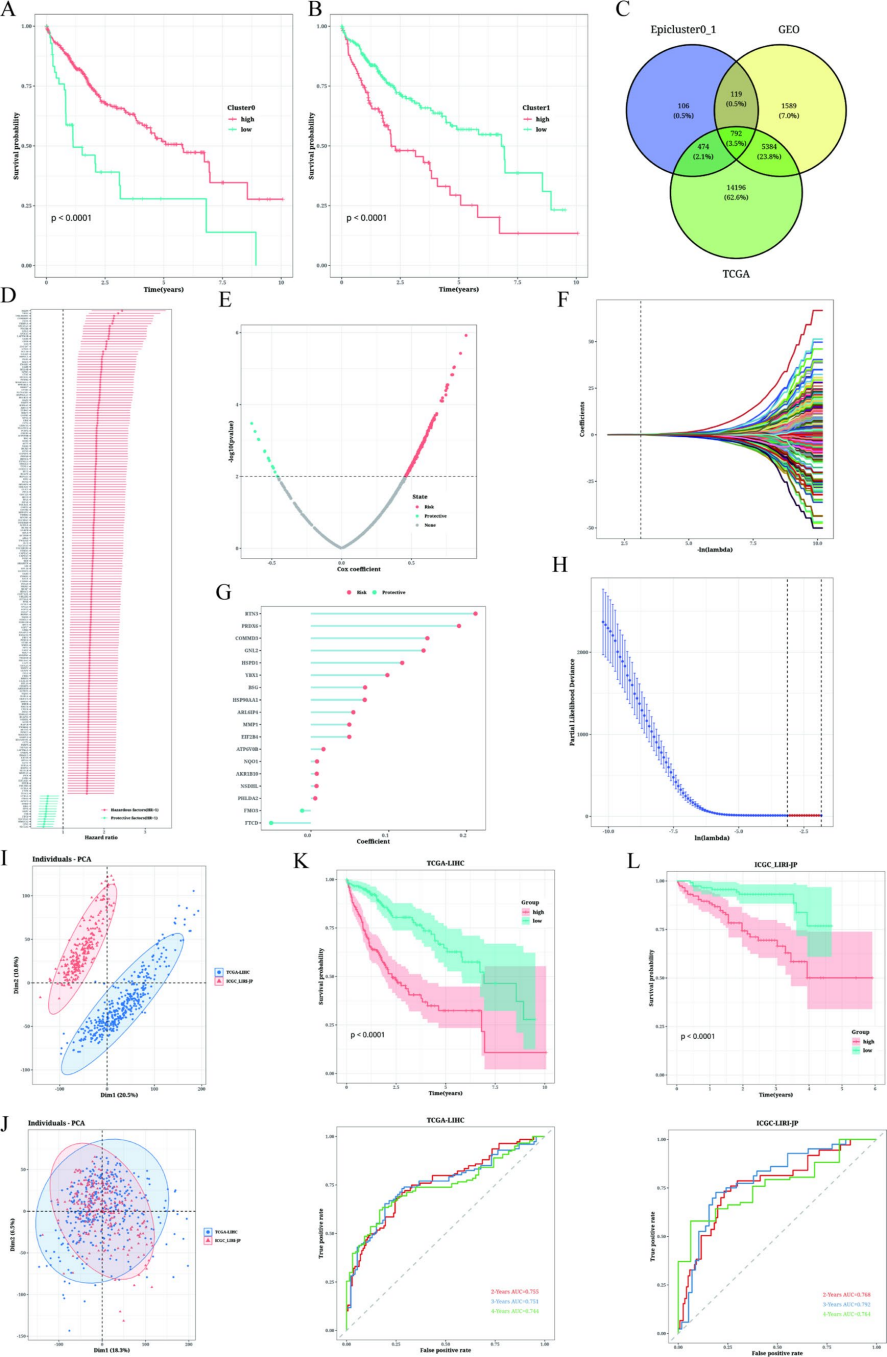
Prognostic model establishment and evaluation results. **A**, **B** Effect of cluster 0 and 1 abundance on survival. **C** Venn diagram showing the overlapping genes of Epicluster0_1 with GEO and TCGA cohorts. **D** Forest plot displaying the results of univariate COX analysis. **E** Volcano plot showing differentially up-regulated and down-regulated genes. **F**, **G** LASSO regression used to select important prognostic genes. **H** Distribution of model gene coefficient values. **I**, **J** Assessment of batch effects in TCGA and ICGC datasets. **K**, **L** Difference in survival rate and time ROC curve between high-risk group and low-risk group in TCGA set and ICGC dataset

Utilizing the ssgsea algorithm, we assessed the abundance of marker genes associated with Cluster 0–1 in TCGA samples. By comparing the survival outcomes between high and low abundance, we observed that Cluster 0, with high abundance, exhibited a higher survival rate, while Cluster 1 showed the opposite trend (Fig. 5A, B). These results were consistent with mentioned observation of higher EMT and aggressiveness in the cluster 1. Through the identification of intersection genes in TCGA, GEO, and Cluster 0–1, we identified a total of 792 marker genes of high expression and related to the grouping of epithelial cell subsets (Fig. 5C). Subsequently, the TCGA dataset was used to construct a prognostic model, and 202 prognostic genes were identified using univariate COX analysis (p < 0.01). These results were visualized using forest plots, which displayed 13 protective factors and 189 risk factors (Fig. 5D). To establish the prognostic model, LASSO-Cox regression analysis was performed, resulting in a model containing 18 genes (Fig. 5E–H). Figure 5I highlighted the presence of significant batch effects in the TCGA dataset and the ICGC dataset, which was diminished for subsequent analysis (Fig. 5J). Survival analysis demonstrated that the high-risk group in the TCGA cohort had significantly worse prognosis than the low-risk group, and this observation was verified in the ICGC cohort as well. Furthermore, the prognostic model exhibited good predictive performance for LIHC patient prognosis, as evidenced by the ROC curve evaluation (AUC value higher than 0.75 at various time points in both training and external cohorts, Fig. 5K, L).

### Immune infiltration in the LIHC patients of different risk values

**Fig. 6:**
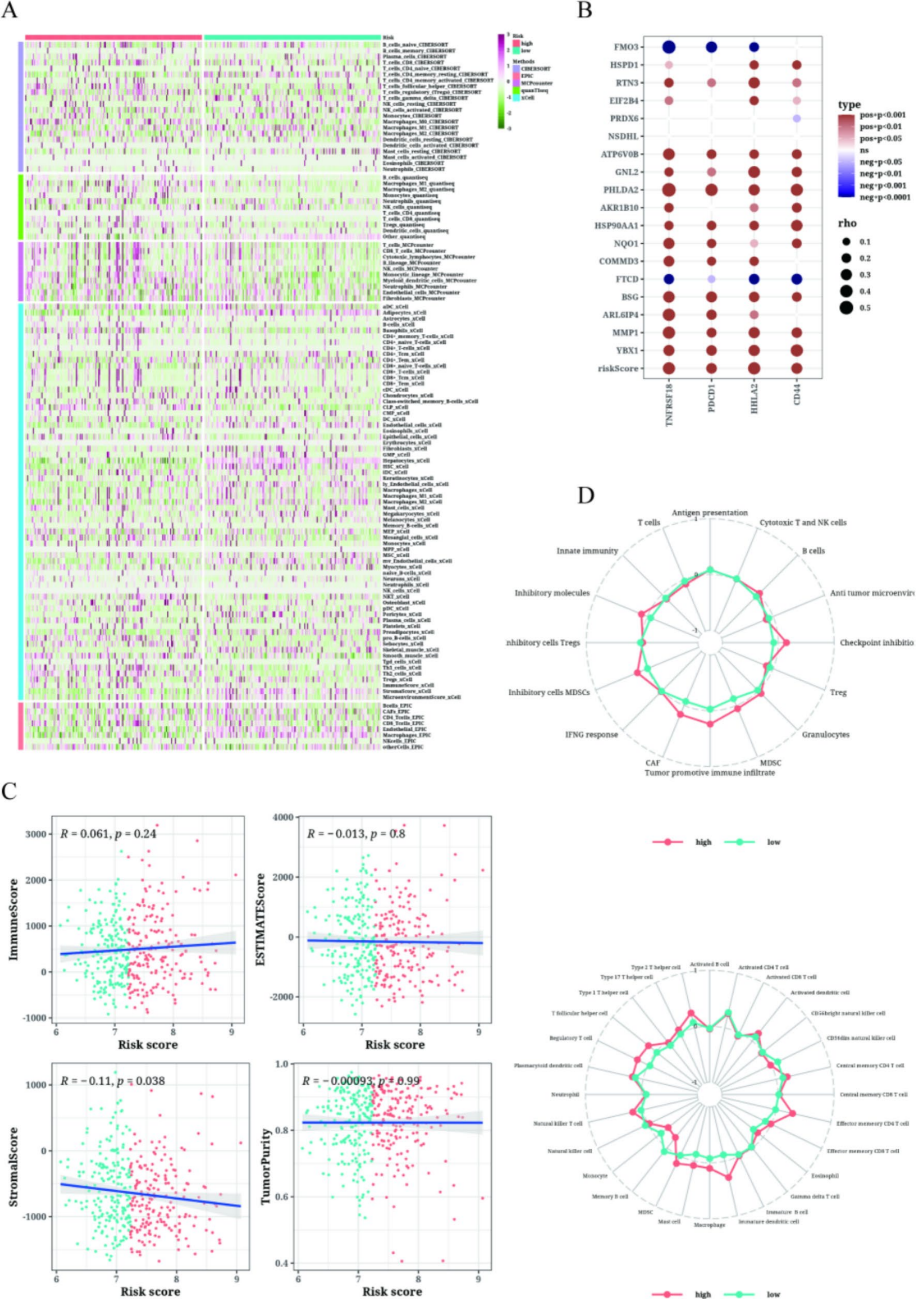
Results of immune infiltration analysis. **A** Heatmap showing the differences in immune cell infiltration between the two risk groups using five algorithms. **B** Bubble plot displaying the correlation between risk score and expression of model genes and immune checkpoints. **C** Scatter plot illustrating the correlation between risk score and matrix score, immune score, ESTIMATE score, and tumor purity. **D** ssGSEA enrichment analysis comparing immune cell infiltration and immune-related pathways in the high-risk group and low-risk group using radar charts

Figure 6A presented a heat map utilizing five different methods to assess immune cell infiltration levels in the high-risk and low-risk groups. The results indicated that immune cell infiltration was more pronounced in the high-risk group, which was featured with inferior prognosis. Additionally, Fig. 6B examined the association between CD44, HHLA2, PDCD1, TNFRSF18, and the risk score, as well as several modeling genes. The results revealed a significant correlation between TNFRSF18 and the risk score. Correlation analysis demonstrated a significant negative correlation between the risk score and the matrix score, while no significant correlation was observed with the other analysis (Fig. 6C). To evaluate the correlation between immune cell infiltration, immune-related pathways, and risk values between the high-risk and low-risk groups, we employed the ssGSEA method. The results indicated that immune cell infiltration in the high-risk group was positively correlated with the risk value and was higher compared to the low-risk group. Additionally, the high-risk group exhibited stronger correlations in several immune-related pathways (Fig. 6D).

### Analysis of TMB and immunotherapy

**Fig. 7:**
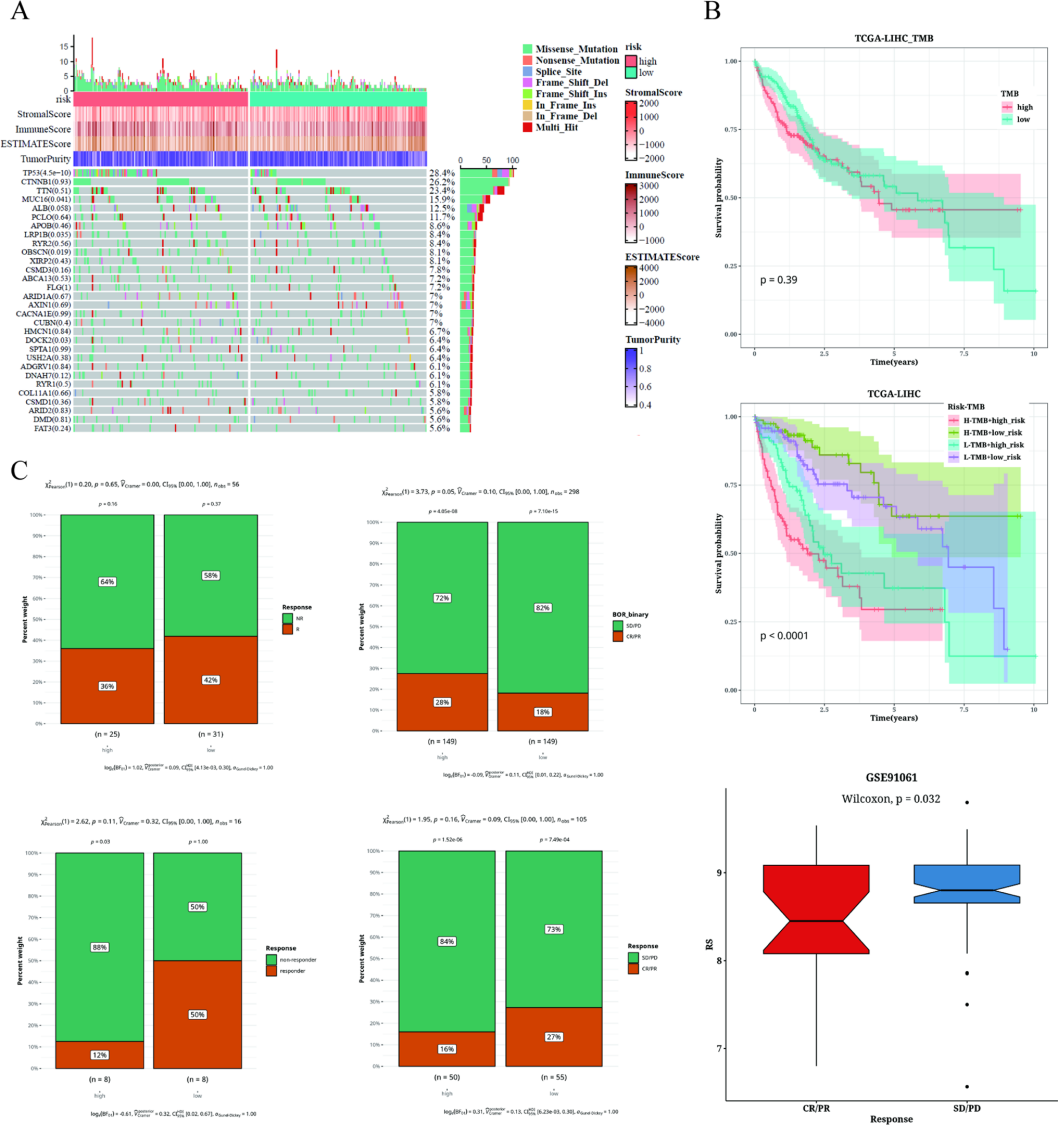
TMB and immunotherapy analysis results. **A** Waterfall plot depicting the differences in liver cancer mutation genes between high-risk and low-risk populations. **B** Survival curves showing survival differences between different subgroups. **C** Analysis of immunotherapy data composition to assess the association between risk values and immunotherapy responses

The waterfall diagram in Fig. 7A illustrated the representative gene mutations in the high-risk and low-risk groups. It is observed that TP53, CTNNB1, TTN, MUC16, and ALB are the five genes with the highest mutation frequency, with TP53, MUC16, and other genes demonstrating significant differences in mutation rate between the two groups. Notably, the heatmap indicates that there is no significant visual difference in TMB between the high-risk and low-risk groups. However, when patients were stratified based on TMB levels, it was found that the prognosis of the high TMB group was not significantly worse or better than that of the low TMB group. Further stratifying patients based on risk score and TMB revealed interesting findings in Fig. 7B. Specifically, the prognosis was observed to be the most unfavorable in the high TMB group and the high-risk group. In cohorts receiving immunotherapy (GSE91061, IMvigor210, GSE126044, and GSE35640), comparative analysis showed that most patients in the low-risk group exhibited significantly higher proportions of treatment responders compared to the high-risk group, with the exception of IMvigor210. We further compared the risk values between the response group and the non-response group in the GSE91061 dataset and found that the risk value of the non-response group was significantly higher than that of the response group (Fig. 7C).

### Enrichment analysis and drug sensitivity analysis

**Fig. 8:**
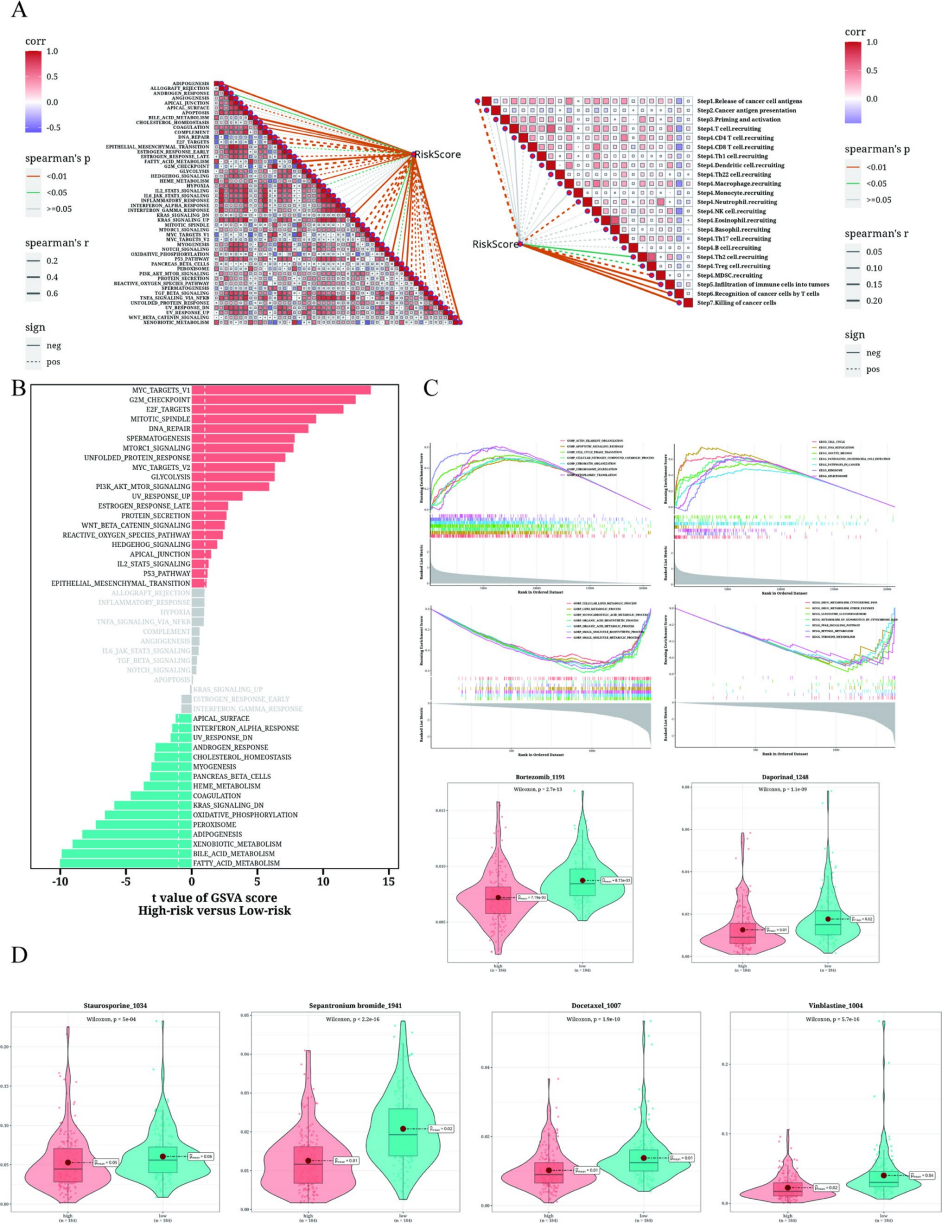
Enrichment analysis and drug sensitivity analysis results. **A** Relationship between risk score and tumor immune cycle steps and marker gene sets. **B** GSVA enrichment analysis showing enrichment of marker gene set between high-risk group and low-risk group. **C** GSEA enrichment analysis displaying enrichment of different genes in GO and KEGG pathways in different risk groups. **D** Box plots comparing the sensitivity of high-risk group and low-risk group to six chemotherapeutic drugs

A comprehensive correlation analysis was conducted to assess the relationship between the risk score and the cancer associated pathways. The findings revealed a significant positive correlation between the risk score and most cancer associated pathways. Notably, a positive correlation was observed between the risk score and KRAS signaling down-regulation and peroxisome (Fig. 8A). To further evaluate the differences in signaling pathways among different risk groups, the marker gene set was used. The enrichment analysis showed that pathways such as MYC/E2F_TARGETs were predominantly enriched in the high-risk group, while pathways related to lipid metabolism were enriched in the low-risk group (Fig. 8B). GSEA analysis identified that cell cycle and lipid metabolism were highly differentially enriched in the high and low risk LIHC groups (Fig. 8C). Additionally, the oncopredict R package was utilized to identify potential effective chemotherapeutic drugs for different risk groups. The analysis revealed six drugs, including Bortezomib_1191, Docetaxel_1007, Daporinad_1248, Sepantronium bromide_1941, Vinblastine_1004, and Staurosporine_1034, which exhibited potential efficacy as anti-tumor drugs in different risk groups (Fig. 8D).

### Validation analysis of model genes

**Fig. 9:**
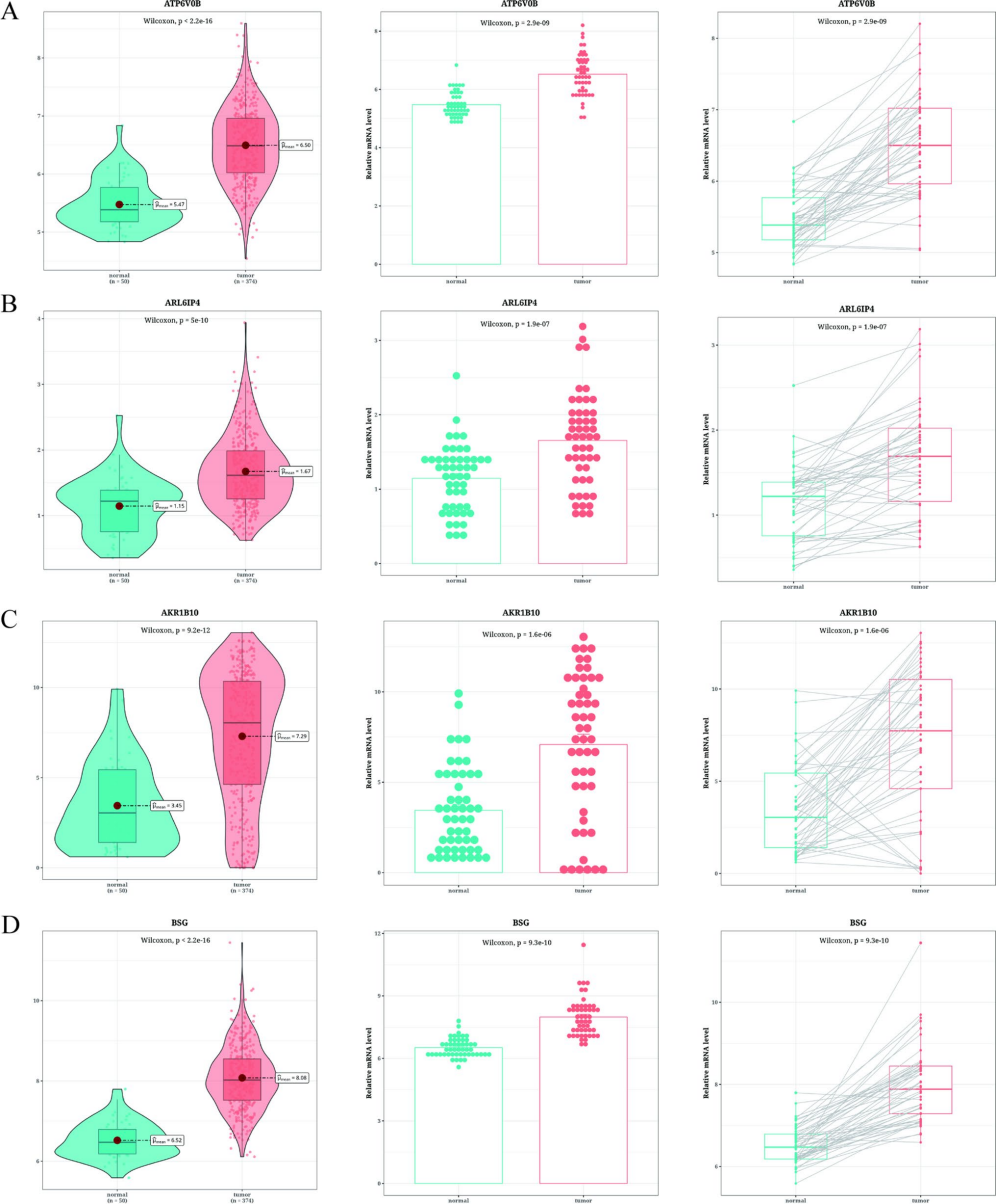
Validation analysis results of model genes. **A**–**D** Box plots showing the differential expression of AKR1B10, ARL6IP4, ATP6V0B, and BSG in TCGA tumors and normal tissues. Gene expression of these model genes in 50 pairs of cancer and para-cancerous samples

In the TCGA dataset, the expression levels of AKR1B10, ARL6IP4, ATP6V0B, and BSG were significantly up-regulated in the tumor samples. To further validate these four model genes, paired tumor and para-cancerous samples in the TCGA data were analyzed, further demonstrating significant up-regulation in tumor tissues (Fig. 9A–D).

## Discussion

This study examined the immune landscape and intratumor heterogeneity of LIHC and identified potential targets for immunotherapy. Using transcriptome data, the samples were grouped into 23 clusters, with most being malignant epithelial cells. Analysis revealed the differentiation states, with cluster 1 being in the terminal state and showing higher aggressiveness and EMT scores. RBP4+ tumor cells were associated with hypoxia processes and extensive cell-to-cell communication. A prognostic model was established, and immune infiltration analysis showed higher infiltration in the high-risk group. TP53 mutation rates differed significantly between the two risk groups. Validation confirmed the up-regulation of model genes in tumor tissues.

TP53 is a well-studied tumor suppressor gene, frequently mutated in various cancers, including LIHC. Previous research has established that TP53 mutations contribute to tumorigenesis by disrupting cell cycle regulation and promoting genomic instability. Studies have shown that TP53 mutations are associated with poor prognosis and increased metastatic potential in multiple cancer types. The impact of TP53 mutations on intercellular communication and EMT within these subgroups remains underexplored. Our study addresses some of these gaps by analyzing TP53 mutation rates in specific epithelial cell subgroups, identifying significant differences between high-risk and low-risk groups. Future research should aim to validate our findings experimentally and explore the mechanisms by which TP53 mutations influence cell-to-cell communication and EMT in HCC. By addressing these gaps, we can further elucidate the role of TP53 in cancer progression and improve therapeutic strategies.

Our study offers insights into LIHC heterogeneity and potential therapeutic targets, and introduces a prognostic model with promising effectiveness. A more pronounced immune cell infiltration was observed in the high-risk group, which was featured with inferior prognosis, suggesting possible cytotoxic cells anergy state. Liver cancer, specifically HCC, is characterized by a dysfunctional immune response. The imbalance between CD4+ and CD8+ cells promote immune tolerance and leads to a poor prognosis. Moreover, HCC cells exhibit lower expression of tumor antigens, resulting in reduced T-cell activation and impaired tumor infiltration. Consequently, the immune system is unable to effectively control tumor growth, leading to worse clinical outcomes [9]. In addition, the compromised function of NK cells and insufficient tumor-infiltrating lymphocytes further contribute to HCC progression. These interconnected factors emphasize the crucial role of addressing immune dysfunction in the development of innovative therapeutic strategies for HCC [10].

In our study, RBP4+ tumor cells were highly enriched with hypoxia process and intensive cell-to-cell communication. RBP4+ tumor cells displayed the highest communication pattern along different types of LIHC malignant cells, showing intensive interactions with the endothelial cells, myeloid cells and CAFs. RBP4, known to be an adipokine associated with hyperinsulinemia and type II diabetes in obese individuals, has previously been identified as a serum marker for multiple cancers, including breast, colorectal and ovarian cancers [11, 12]. RBP4 was overexpressed in ovarian cancer cells, similar to adipose tissues. Overexpression of RBP4 promoted cancer cell migration and proliferation. Molecularly, RBP4 induced the expression of cancer progression factors MMP2 and MMP9. Moreover, RhoA/Rock1 pathway activation and upregulation of CyclinD1 were involved in RBP4-induced ovarian cancer cell migration [13]. The transmembrane protein STRA6 acts as a vitamin A transporter and cytokine receptor, activated by vitamin A-bound RBP4. In a xenograft mouse model of colon cancer, STRA6 activation triggers a JAK2-STAT3 signaling cascade, promoting tumorigenesis. The expression of RBP4 and STRA6 is linked to poor prognosis in oncology. Downregulating STRA6 or RBP4 in colon cancer cells reduces the proliferation of cancer stem cells and sphere formation [14]. However, few studies were conducted to study the role of RBP4 in the LIHC carcinogenesis. Intriguingly, RBP4 was also reported to function as a biomarker for cardiovascular diseases risk, suggesting a potential role of metabolic dysregulation of RBP4, which could be associated with advanced glycolysis rate in the RBP4+ tumor cells.

Cluster 1 subgroup of malignant cells exhibits significantly higher Aggressive scores compared to the other cell subsets. Moreover, Cluster 1 displays markedly higher EMT scores than Cluster 0 and Cluster 2, indicating a more pronounced migratory ability of LIHC epithelial cells within the Cluster 1 subgroup. There were a plethora of evidence linking EMT with LIHC prognosis and aggressiveness. The expression level of ADGRE5 was strongly associated with EMT in various types of cancer. ADGRE5, an adhesion regulating receptor, was differentially expressed in multiple cancers and was significantly associated with patient survival outcomes. Higher levels of ADGRE5 were associated with worse prognosis in both lung squamous cell carcinoma and LIHC [15]. In another study studying LIHC metastasis, IGFBP2, a protein involved in cell growth and metastasis, was specifically expressed in the plasma exosomes of LIHC patients. Higher levels of IGFBP2 in these exosomes were associated with a poor prognosis in LIHC patients. As regard to the underlying mechanism, IGFBP2 in these exosomes activated the ERK signaling pathway, which triggered EMT [16]. GINS1 was significantly upregulated in HCC tissues and cell lines, particularly in those with vascular invasion and high metastatic potential. As a well-established EMT regulator, ZEB1 was shown to be vitally implicated in GINS1-induced cancer progression through promoting EMT and tumor metastasis via β-catenin signaling [17]. MiR-557 expression was decreased in HCC tissues and cell lines, and this decrease was associated with recurrence and metastasis of HCC. Overexpression of miR-557 inhibited cell proliferation, migration, invasion, and EMT in vitro, and suppressed tumor growth in vivo. The researchers also identified RAB10 as a direct target of miR-557, and showed that miR-557 regulates the Wnt/β-catenin pathway through RAB10 [18]. In summary, the expression levels of EMT regulating factors were closely associated with the development and progression of HCC.

A potential limitation of this study is the reliance on publicly available datasets, which may introduce biases related to sample collection and processing methods. Additionally, the prognostic model, although effective across multiple cohorts, may require additional refinement and testing in diverse patient populations to ensure its generalizability and clinical applicability.

## Conclusion

A prognostic model was established based on HCC malignant cell associated gene signature, displaying decent prognosis guiding effectiveness in the multiple cohorts. The study provided comprehensive insights into the heterogeneity and potential therapeutic targets of LIHC.

## Data Availability

The data could be obtained by contacting the corresponding author.
